# Use of asthma drugs and prevalence of molar incisor hypomineralization

**DOI:** 10.1111/ipd.12655

**Published:** 2020-04-28

**Authors:** Pia Wogelius, Jakob Hansen Viuff, Dorte Haubek

**Affiliations:** ^1^ Aalborg Municipal Dental Service Aalborg Denmark; ^2^ Department of Clinical Epidemiology Aarhus University Hospital Aarhus N Denmark; ^3^ Section for Pediatric Dentistry Department of Dentistry and Oral Health Aarhus University Aarhus C Denmark

**Keywords:** anti‐asthmatic agents, asthma, child, cross‐sectional studies, dental enamel, epidemiology, molar

## Abstract

**Background:**

Asthma and molar incisor hypomineralization (MIH) are common diseases among children and have been suspected to be associated with each other.

**Aim:**

To examine the association between asthma or the use of asthma drugs and the prevalence of MIH.

**Design:**

In a population‐based cross‐sectional study, we recorded MIH in 9‐year‐old children in Aalborg Municipality, Denmark, born in the year 2000. We used a unique 10‐digit civil personal number to link data on MIH to population‐based medical register data. The exposure was inhaled asthma medication from birth date until date of dental examination. The outcome was the overall prevalence of MIH according to use of asthma medication. Odds ratios (OR) of having MIH were adjusted for gender, use of antibiotics and amoxicillin, maternal smoking, pre‐ and perinatal complication, and hospital admissions.

**Results:**

We examined 1837 children, of which 542 (29.5%) had one or more molar(s) with MIH. The adjusted odds ratio of having MIH was 0.95 (95% CI: 0.60‐1.51) among children with prescriptions of inhaled asthma medication.

**Conclusion:**

In this study, where the results have been adjusted for confounding, we found no association between use of inhaled asthma medication and the prevalence of MIH.

## 1 INTRODUCTION

Demarcated opacities are often seen in first permanent molars and frequently associated with affected incisors. When the enamel hypomineralization is present in one or more first permanent molars, the condition is classified as molar incisor hypomineralization (MIH).^1^, ^2^, ^3^, ^4^, ^5^ The prevalence of MIH is examined in several studies worldwide, and prevalence between 2.4% and 44% have been reported.^6^ The studies were, however, carried out under un‐standardized examination settings, including, for example, different classification systems, different light sources, and tooth drying protocols. Altogether, this might possibly explain the wide variation. A previous population‐based study from Denmark among 647 6‐ to 8‐year‐old children with fully erupted first permanent molars showed that 37.3% (95% CI: 33.6%‐41.0%) had MIH and 6.3% (95% CI: 4.7%‐8.5%) had one or more first permanent molar(s) with post‐eruptive enamel breakdown (PEB) due to hypomineralization.^7^ Among the 6‐year‐olds, the prevalence of PEB was 4.7% (95% CI: 2.4%‐8.9%), and among the 8‐year‐olds, the prevalence was 8.2% (95% CI: 5.4%‐12.4%), indicating that the development from severe and profound MIH with an intact enamel surface to enamel breakdown takes time. A considerable proportion of children needs dental treatment due to MIH.^8^, ^9^, ^10^

Silva and coworkers published a literature review in 2016 on the aetiology of MIH.^11^ The literature showed evidence to some extent of an association between illnesses and drug use in early childhood and an increased prevalence of MIH, while less evidence of associations with prenatal and perinatal exposures, such as preterm birth, caesarian delivery, or birth complications, was found.

Asthma and MIH are frequent diseases among children, and because the frequently used corticoid‐steroid treatment in children and teenagers^12^ has an effect on bone growth,^13^ it may affect the tooth mineralization process as well and hypothetically cause MIH.

Few studies have examined the association between asthma and MIH. Furthermore, the studies on the topic differ according to design and size of the study population. Therefore, common agreement based on previous results is difficult to obtain.^14^, ^15^, ^16^, ^17^, ^18^, ^19^, ^20^, ^21^, ^22^, ^23^

Previously, the association between the use of asthma drugs and the risk of MIH was examined in the above‐mentioned 647 Danish children being 6‐8 years of age.^18^ We used register‐based information on the use of asthma drugs in order to eliminate recall bias, and we found an increased, but not significant, risk of MIH with PEB in children with prescriptions of asthma drugs between the age of 0 and 3 years of age (OR 2.42; 95% CI: 0.70‐7.43). Furthermore, a limitation in our first study was the lack of control of confounding. In order to explore the association between asthma and MIH further, we conducted a cross‐sectional study with a higher number of children included and addressed the possibility of confounding by including register‐based information on possible confounders. The aim of this study was to examine the association between asthma or the use of asthma drugs and the prevalence of MIH.

## 2 MATERIALS AND METHODS

### 2.1 Design and study population

This study is a population‐based cross‐sectional study conducted in Aalborg Municipality, Denmark. The municipality consists of one large city, Aalborg, several smaller towns, and rural areas. In 2012, the municipality had 203 475 inhabitants, of which 12 348 were children below the age of 15 years (Source: National Board of Health, Denmark). In Denmark, dental service is mandatory and free of charge for all children below the age of 18 years. The service is mostly conducted at public clinics, although private dental care is a possibility by paying 35% of the fee. In Aalborg Municipality, almost all children use the 10 public clinics (approximately 99.8% of all inhabitants below 15 years of age use the public clinics). The study included all children born in the year 2000 (n = 2002) attending the public clinics. Of the 2002 included children, 1837 (91.8%) were examined for MIH during the regular visits at the public dental clinics. The observational study conforms to the STROBE (Strengthening the Report of Observational Studies in Epidemiology) guidelines. First, we made a dental examination of the included children. Afterwards, we collected data on exposure(s) and confounders from national public registries. All data were linked by using the unique civil registry number; a 10‐digit number assigned to Danish residents since year 1968 and that encodes residents gender and date of birth.^24^ The numbers are used in all Danish registries, permitting unambiguous individual‐level linkage of data from all sources used in this study and allowing identification of the mothers of all children.

### 2.2 Ethics

The Danish Data Protection Agency approved the study (File # 2016‐41‐4845).

### 2.3 Information on exposure

As part of a national tax‐supported health programme, all pharmacies in Denmark are equipped with a computerized accounting system by which data on sold medical products are sent to the Danish National Health Service. The programme refunds part of the costs associated with the purchases of the prescribed drugs, including asthma drugs. The accounting system provides key data on prescriptions for refundable drugs to the Danish National Prescription Registry.^25^, ^26^ We collected information on: (a) type of drug according to the Anatomical‐Therapeutic‐Chemical (ATC) classification system, (b) date of purchasing the prescription drug, and (c) the patient's civil registry number. Asthma drugs were classified as: (a) inhaled β_2_‐agonists, (b) oral β_2_‐agonists, and (c) inhaled corticosteroids. We established a complete prescription history for all children included in this study. Asthma drugs were only included if the user had at least two prescriptions. The cut‐off point on at least two prescriptions was decided based on the following two reasons: (a) asthma drugs are sometimes prescribed in the diagnostic process and intake of the drugs are not continued, if no improvement of the condition is seen, and (b) some parent do not comply and discontinue purchase the drugs after the first prescriptions.

### 2.4 Information on confounders

#### 2.4.1 Birth outcomes and neonatal information

We collected data on birth outcomes and neonatal information from the Danish Medical Birth Registry (DMBR) and the Danish National Registry of Patients (DNRP).^26^, ^27^ Registration to DMBR is mandatory for all maternity units in Denmark. The information collected from the DMBR was (a) maternal smoking, (b) intrauterine asphyxia, (c) gestational age, (d) birthweight, (e) signs of asphyxia after birth, and (f) number of admissions days on a neonatal department. Maternal smoking was self‐reported. Low birthweight was defined as less than 2500 kg, and the variable for preterm birth included children born before 37th week of gestational age.

The DNRP contains information on all inpatient and outpatient hospital contacts in Denmark, including dates of admission and discharge and one or more discharge diagnoses coded by physicians according to the *International Classification of Diseases*. The International *Classification of Diseases, Tenth Revision*, was used after 1997. From the DNRP, we collected information on all asthma diagnoses (DJ45) from birth until the age of 9 years of the population.

#### 2.4.2 Attention Deficit Hyperactivity Deficit (ADHD) drugs and antibiotics

We collected information on ADHD drugs and antibiotics from the Pharmacological Prescription Database. Antibiotics were divided into frequency of prescription: ‘No’, ‘1‐2 times’, and ‘more than 2 times’. The first prescription of ADHD drugs was discarded, and only subsequent prescriptions were included. The number of children with ADHD drug use was low (Table 1), and we therefore left out information on ADHD drugs from further analyses. This number of children did not differ, if we included first prescription, thus all children receiving ADHD drugs received more than one prescription.

**TABLE 1:** Characteristics of 2002 children included and 1837 dentally examined children

Variable	Included children	Examined children
	n (%)	n (%)
Gender		
Female	980 (49.0)	896 (48.8)
Asthma drugs		
No^a^	1263 (63.1)	1158 (63.0)
Peroral β‐2 agonists only^b^	537 (26.8)	489 (26.6)
Inhaled β‐2 agonists only^b^	18 (0.9)	17 (0.9)
Both inhaled β‐2 agonists and steroids	50 (2.5)	47 (2.6)
Antibiotics		
No	157 (7.8)	143 (7.8)
1‐2 prescriptions	428 (21.4)	392 (21.3)
>2 prescriptions	1417 (70.8)	1302 (70.9)
Amoxicillin	1269 (63.4)	1166 (63.5)
ADHD drugs^b^	37 (1.8)	32 (1.7)
Maternal smoking^c^	447 (22.3)	401 (21.8)
Birth outcomes		
Preterm (<37 wk)^c^	123 (6.1)	109 (5.9)
Low birthweight (<2500 g)^c^	96 (4.8)	85 (4.6)
Asphyxia^c^	168 (8.4)	159 (8.7)
Neonatal hospitalization^c^	196 (9.8)	179 (9.7)
Hospital admissions with asthma (DJ45)	126 (6.3)	113 (6.2)

^a^ Not including only one prescription.

^b^ At least two prescriptions.

^c^ Missing information on maternal smoking (n = 127), preterm (n = 123), low birthweight (n = 118), asphyxia (n = 87), and neonatal hospitalization (n = 87).

### 2.5 Information on outcome

#### 2.5.1 Training and calibration of examiners

One clinician at each of the 10 clinics attended the group of examiners. At a preliminary meeting with the examiners, we presented the study and the examination criteria developed by Weerheijm et al^28^. The group of examiners was trained as done in our previous study,^7^ meaning in the use of the same scoring sheet, written description material, and photographic examples of stages of the enamel hypomineralization. Furthermore, a calibrating meeting was organized, and we invited three nine‐year‐old children with MIH. All examiners and PW examined the same children one time and discussed the examination criteria. One of the coauthors (DH) supervised this meeting. Two of the children was not able to cooperate to be examined by all examiners and was examined by 7 and 10 examiners, respectively. All examiners agreed on the MIH status of the three examined children at patient level, whereas some disagreement occurred at tooth level. During the examination period, all examiners were welcomed to mail clinical photographs or questions to PW in case of doubt about the severity of the MIH status. The written description material recommended the examiners to choose the less severe diagnosis in case of doubt of presence of opacity or not.

#### 2.5.2 Collection of dental data

During 2009, the trained examiners examined all included children born in 2000 during the regularly yearly dental visits. The results of the MIH examination were noted by hand on the scorings sheets, collected, and afterwards sent to PW.

### 2.6 Statistics

The outcome, MIH was included as a best‐case scenario, thus children with missing information on one or more first permanent molars (n = 23) was regarded as not having MIH. This decision was made not to overestimate the prevalence of MIH. We conducted a sensitivity analysis dividing MIH into yellow opacities or PEB (white only was excluded) and white only (yellow opacities and PEB were excluded), and the results did not differ substantially.

Crude and adjusted odds ratios (OR) were calculated using logistic regression. Furthermore, OR was adjusted for gender, use of antibiotics and amoxicillin, maternal smoking, pre‐ and perinatal complications, and hospital admission with asthma diagnose (DJ45). *P*‐values less than .05 were considered statistical significant, but both estimates and 95% confidence levels support the conclusions. Statistical analysis and data management were performed using SAS software version 9.4 (SAS Institute Inc).

## 3 RESULTS

The initial training and calibration session showed 100% agreement regarding the MIH diagnoses at patient level, while the agreement at tooth level differed to some extent. The following calibration sessions showed the same tendency.

The study population consisted of 2002 children, born in the year 2000, of which 1837 (91.7%) were clinically examined. The remaining children were not examined for the many reasons, that is, moving outside the municipality, missing the appointment, the individual interval between two routine examinations was longer than one year, various circumstances related to scheduling routines, unforeseen emergency visits, or lack of attention from the dental staff. The examined children did not differ from the included ones (Table 1).

A number of 1158 children had no prescriptions of asthma medication, and the remaining 37.0% had received one or more prescription of different kind of asthma medication. The most common type of asthma medication prescribed was peroral β‐2 agonists, which was prescribed at least twice to 489 (26.6% of the children, who underwent a dental examination). The number of children who received prescription of both inhaled β‐2 agonists and inhaled corticosteroids was 47 (2.6%).

The overall prevalence of MIH was 29.5%, of which 352 (19.2%) had MIH with white opacities only, 60 (3.3%) had MIH with yellow ± white opacities, and 130 (7.1%) had MIH with post‐eruptive breakdown ± yellow or white opacities.

The crude OR of having MIH in children with at least two prescriptions of both inhaled beta‐2 and steroid was 1.48 (95% CI: 0.81‐2.70) (Table 2) compared with children with no asthma prescriptions. In addition, data showed elevated odds of having MIH among boys compared with girls. No other factor was significantly associated with an elevated or reduced odds. Adjusting for gender, use of antibiotics, including amoxicillin, maternal smoking, pre‐ and perinatal complications, and hospital admission with asthma diagnose (DJ45), we calculated the adjusted OR of having MIH in children with at least two prescriptions of both inhaled beta‐2 and steroid to be 0.95 (95% CI: 0.60‐1.51). We made a separate analysis of the odds of having MIH with yellow opacities or post‐eruptive breakdown, and in this case, the adjusted OR was 0.80 (95% CI: 0.38‐1.69).

**TABLE 2:** Crude odds ratios (OR) of molar incisor hypomineralization (MIH) in relation to asthma drug use, gender, use of antibiotics, maternal smoking, birth outcome, and hospitalization due to asthma

Predictor	Children	Children with MIH		Crude OR (95% CI)
	n	n	Prevalence % (95% CI)	
Overall	1837	542	29.5 (27.5‐31.6)	
Gender				
Female	896	239	26.7 (23.9‐29.6)	0.77 (0.63‐0.94)
Male	941	303	32.2 (29.3‐35.2)	
Asthma drugs				
No^a^	1158	342	29.5 (27.0‐32.2)	
Peroral ß2‐agonists only^b^	489	141	28.8 (25.0‐33.0)	0.97 (0.77‐1.22)
Inhaled ß2‐agonists only^b^	17	4	23.5 (8.5‐46.7)	0.73 (0.24‐2.27)
Both inhaled ß2‐agonists and steroids	47	18	38.3 (25.4‐52.6)	1.48 (0.81‐2.70)
Antibiotics				
No	143	47	32.9 (25.6‐40.8)	
1‐2 prescriptions	392	113	28.8 (24.5‐33.5)	0.83 (0.55‐1.25)
>2 prescriptions	1302	382	29.3 (26.9‐31.9)	0.85 (0.59‐1.23)
Amoxicillin				
No	671	202	30.1 (26.7‐33.7)	
Yes	1166	340	29.2 (26.6‐31.8)	0.96 (0.78‐1.18)
Maternal smoking^c^	401	118	29.4 (25.1‐34.0)	0.97 (0.76‐1.24)
Birth outcomes				
Preterm (<37 wk)^c^	109	33	30.3 (22.3‐39.3)	1.02 (0.67‐1.55)
Low birthweight (<2500 g)^c^	85	28	32.9 (23.6‐43.4)	1.16 (0.73‐1.85)
Asphyxia^c^	159	48	30.2 (23.5‐37.6)	1.03 (0.72‐1.46)
Neonatal hospitalization^c^	179	51	28.5 (22.3‐35.4)	0.94 (0.67‐1.32)
Hospital admission with asthma (DJ45)	708	208	29.4 (26.1‐32.8)	1.28 (0.86‐1.91)

^a^ Not including only one prescription.

^b^ At least two prescriptions.

^c^ Missing information on maternal smoking (n = 113), preterm (n = 107), low birthweight (n = 105), asphyxia (n = 77), and neonatal hospitalization (n = 77).

## 4 DISCUSSION

The present population‐based study among 9‐year‐old Danish children showed no association between use of inhaled asthma medication and an increased prevalence of MIH. Our study is the first to control for possible confounders, and we used registry‐based information on asthma medication and on confounders.

Our data analyses showed no differences between the dropouts and the examined children regarding exposure and confounders. However, we do not know the MIH status of the dropouts. The prevalence of MIH might differ among the dropouts compared to the examined children. This will, however, not cause selection bias unless the association between asthma and MIH differs among the dropouts and the examined group.

We trained and calibrated the examiners, who were all experienced dentists with full‐time employment at the municipal dental service in Aalborg, Denmark. MIH is a condition with clinical manifestation ranging from small changes to extensive enamel breakdown.^28^ The milder the lesion(s) are, the more likely they will be overseen or confused with initial stages of dental caries or other types of dental anomalies. Especially when the enamel defects are located at the approximal surface, it is possible to overlook the condition. Therefore, we performed separate analyses according to the severity of the MIH and found no increased risk of MIH in children with use of asthma medication, regardless of type, and severity of MIH.

The validity of the MIH diagnose is not examined in other studies. Our results showed high agreement between the examiners findings at patient level in children with many teeth with demarcated opacities, while it varied at the tooth level. Consequently, misclassification of MIH may occur in this study. However, this misclassification is most likely equally distributed in children with and without asthma and therefore not expected to affect the estimated odds ratios. The examiners were not aware of the asthma condition of the examined children, and therefore, any misclassification of MIH will be equally distributed between children with and without asthma and most likely not bias the relative result.

A strength in our study is that our data on exposure were register‐based. Thereby, we avoided recall bias. The only self‐reported data in our study were the information on maternal smoking status. The validity on self‐reported smoking information in Danish women is not known. We cannot exclude the possibility that maternal smoking is linked to both asthma disease and MIH in the offspring. Therefore, our study might be biased in two ways due to maternal smoking: (a) recall bias and (b) confounding bias. Although we adjusted for several possible confounding factors, unknown confounders might still have caused biases. Recently, Nørrisgaard and coworkers found in a Danish Child Asthma Cohort that high supplementation with vitamin D during pregnancy reduced the prevalence of MIH significantly in the offspring.^29^

In this study, MIH tended to be more prevalent among boys compared with girls. This was in accordance with a previous Danish study.^7^ Although the gender difference is small, it seemed to be enough to confound the crude odds ratios. Other studies have found that the gender difference was opposing.^30^ At present time, there is no consensus on the topic. Furthermore, we would have liked to include information on ethnicity and socioeconomic variables. However, it was not possible for us to get access to this kind of data.

In 2010, we found an association between use of asthma drugs and an increased prevalence of children with more seriously affected molars with MIH.^18^ The association was not statistical significant, and we could not confirm the finding in this study. In our first study on the association between use of asthma drugs and prevalence of MIH, we did not control for confounding and this may explain the difference between our former and this study.

A number of other studies found an increased risk of having MIH in children with asthma, or with asthma drug use, while others found no such an association.^11^ Our study differed in several ways from other studies in being population‐based, using register‐based data, and in controlling for confounding.

In conclusion, the use of asthma drugs does not increase the prevalence of MIH in Danish children, as we found no association between use of inhaled asthma medication and the prevalence of having MIH.

**Why this paper is important to paediatric dentists?**

- Asthma and molar incisor hypomineralization is common diseases among children.
- Use of asthma drugs has been suspected to be associated with molar incisor hypomineralization.
- This study showed no such association and is the first to adjust for confounding.

## CONFLICTS OF INTEREST

The authors declare no conflicts of interest.

## AUTHORS’ CONTRIBUTIONS

PW and DH planned and designed the study. PW drafted the main part of manuscript. JV drafted the statistic section of the manuscript. JV performed all statistical analyses together with Sinna Pilgaard Ulrichsen after meeting held together with PW and DH. All authors adhere to the International Committee of Medical Journal Editor's requirements regarding authorship. All authors critically revised drafts and approved the final manuscript.
